# Prospective validation of the NCI Breast Cancer Risk Assessment Tool (Gail Model) on 40,000 Australian women

**DOI:** 10.1186/s13058-018-1084-x

**Published:** 2018-12-20

**Authors:** Carolyn Nickson, Pietro Procopio, Louiza S. Velentzis, Sarah Carr, Lisa Devereux, Gregory Bruce Mann, Paul James, Grant Lee, Cameron Wellard, Ian Campbell

**Affiliations:** 1 Melbourne School of Population and Global Health, University of Melbourne, Carlton, Victoria 3010 Australia; 2 Cancer Research Division, Cancer Council NSW, Woolloomooloo, NSW 2011 Australia; 3 Lifepool Study, Peter MacCallum Cancer Centre, Melbourne, Victoria 3000 Australia; 4 Breast Service, Royal Women’s and Royal Melbourne Hospital, Parkville, Victoria 3050 Australia; 5 Department of Surgery, The University of Melbourne, Parkville, 3010 Australia; 6 Sir Peter MacCallum Department of Oncology, University of Melbourne, Melbourne, Victoria 3000 Australia; 7 Familial Cancer Centre, Peter MacCallum Cancer Centre and Royal Melbourne Hospital, Parkville, Victoria 3052 Australia; 8 Cancer Genetics Laboratory, Peter MacCallum Cancer Centre, Melbourne, Victoria 3000 Australia

**Keywords:** Breast cancer screening, Validation, Gail model, Breast Cancer Risk Assessment Tool, Machine learning, Invasive breast cancer, Risk stratification

## Abstract

**Background:**

There is a growing interest in delivering more personalised, risk-based breast cancer screening protocols. This requires population-level validation of practical models that can stratify women into breast cancer risk groups. Few studies have evaluated the Gail model (NCI Breast Cancer Risk Assessment Tool) in a population screening setting; we validated this tool in a large, screened population.

**Methods:**

We used data from 40,158 women aged 50–69 years (via the *lifepool* cohort) participating in Australia’s BreastScreen programme. We investigated the association between Gail scores and future invasive breast cancer, comparing observed and expected outcomes by Gail score ranked groups. We also used machine learning to rank Gail model input variables by importance and then assessed the incremental benefit in risk prediction obtained by adding variables in order of diminishing importance.

**Results:**

Over a median of 4.3 years, the Gail model predicted 612 invasive breast cancers compared with 564 observed cancers (expected/observed (E/O) = 1.09, 95% confidence interval (CI) 1.00–1.18). There was good agreement across decile groups of Gail scores (χ^2^ = 7.1, *p* = 0.6) although there was some overestimation of cancer risk in the top decile of our study group (E/O = 1.65, 95% CI 1.33–2.07). Women in the highest quintile (Q5) of Gail scores had a 2.28-fold increased risk of breast cancer (95% CI 1.73–3.02, *p* < 0.0001) compared with the lowest quintile (Q1). Compared with the median quintile, women in Q5 had a 34% increased risk (95% CI 1.06–1.70, *p* = 0.014) and those in Q1 had a 41% reduced risk (95% CI 0.44–0.79, *p* < 0.0001). Similar patterns were observed separately for women aged 50–59 and 60–69 years. The model’s overall discrimination was modest (area under the curve (AUC) 0.59, 95% CI 0.56–0.61). A reduced Gail model excluding information on ethnicity and hyperplasia was comparable to the full Gail model in terms of correctly stratifying women into risk groups.

**Conclusions:**

This study confirms that the Gail model (or a reduced model excluding information on hyperplasia and ethnicity) can effectively stratify a screened population aged 50–69 years according to the risk of future invasive breast cancer. This information has the potential to enable more personalised, risk-based screening strategies that aim to improve the balance of the benefits and harms of screening.

**Electronic supplementary material:**

The online version of this article (10.1186/s13058-018-1084-x) contains supplementary material, which is available to authorized users.

## Background

National guidelines and programmes for universal age-based breast cancer screening were established in many countries following trials showing reduced breast cancer mortality [1–4]. However, increasing evidence on measurable risk factors for breast cancer [5, 6] and growing concern about overdiagnosis [7, 8] and the appropriateness of mammography for women with dense breasts [9, 10] has fuelled interest in more personalised, risk-stratified screening protocols that better optimise the balance of the benefits and harms of screening [11]. A number of countries have established nationally co-ordinated screening programmes. Australia, for example, has a breast cancer screening programme (BreastScreen Australia) offering free biennial mammographies targeted towards women aged 50–74 years (extended from 50 to 69 years in mid-2015) with participation of approximately 55% [12]. Similar programmes have been established in the UK, Canada, Europe, and elsewhere. While risk-stratified screening intervals and more intensive surveillance for high-risk women or women with high mammographic density has been proposed [13], there are no widespread protocols for tailored breast cancer screening in Australia or internationally.

Risk-stratified screening protocols require accurate estimates of risk using data that can be readily obtained by population-based programmes. The Gail model [14–16] is relatively simple, requiring minimal information on the family history of cancer. The original model estimated absolute risk of invasive and in-situ breast cancers [17], and was later modified [18] and incorporated into the National Cancer Institute’s Breast Cancer Risk Assessment Tool (hereafter referred to as the Gail model) and used for predicting invasive breast cancer risk for women without a personal history of breast cancer [19]. The Gail model has performed well on white women residing in the US and Europe [20–22], with poorer performance in women of other ethnic backgrounds, such as African American, Hispanic, Asian, and Pacific Islander women [23–25]. In Australia, the performance of the Gail model has been assessed for high-risk women [26] and women younger than 60 years of age [27].

The *lifepool* cohort comprises 53,800 women recruited since 2010 primarily from the Australian population-based mammography screening programme to facilitate research into breast cancer screening, epidemiology, and genetics. Using data from baseline questionnaires, we generated Gail risk estimates for active breast cancer screening participants in the historical target age range for screening (50–69 years) and compared predicted and observed risk of incident invasive breast cancer. In addition, we evaluated risk estimates from reduced Gail models, assessing the incremental benefit obtained by adding variables to the model in order of diminishing contribution to risk estimation.

## Methods

### Study participants

*Lifepool* commenced recruitment in May 2010, restricted to women aged at least 40 years at enrolment. Up to January 2015, recruitment was primarily through an invitation included in appointment letters for women attending subsequent rounds of screening at the BreastScreen programme based in the Australian state of Victoria (BreastScreen Victoria). Other methods of recruitment were publicity at women’s health events, referrals by participants to friends and family, and inclusion as a research project on the national database Register4 [28] in July 2012. On enrolment, *lifepool* participants complete a detailed ‘baseline’ questionnaire capturing socio-demographic, lifestyle, and health-related information. Further details on the cohort including the questionnaire and other material can be found on-line (http://www.lifepool.org). The *lifepool* cohort is regularly linked to BreastScreen Victoria records and to the Victorian Cancer Registry to update information on the occurrence of any cancer diagnosed within the state of Victoria.

### Data provided for this analysis

Complete questionnaire data were provided for this study for all participants who completed baseline questionnaire data up to 11 September 2016. *Lifepool* also provided linked data comprising: 1) BreastScreen Victoria screening episodes up to 27 June 2017 with information on screening dates and cancer diagnoses (screen-detected or interval cancer, diagnosis date, invasive or in situ); and 2) Victorian Cancer Registry breast cancer diagnoses (date, invasive, or in situ) and, for women with any cancer registration, death records (date, cause of death). *Lifepool* also provided participant withdrawals and ad hoc death notifications and cancer diagnosis outside Victoria. Data provision is described in Additional file 1.

### Statistical analyses

#### Gail scores

Gail risk scores were assigned using the source code available on the National Cancer Institute website [19], which generates the probability of breast cancer for some specified integer year in the future (e.g. 5-year risk), or to a fixed age in years for a study population. To evaluate the Gail model as a potential tool for assessing the risk of future breast cancer following a clear screen, we restricted our analyses to women aged 50–69 years who had had a screening episode with a benign final outcome within ±60 days of completing their baseline study questionnaire (‘reference screen’) and, as per the model’s specification, no personal history of invasive breast cancer, ductal carcinoma in situ (DCIS), or lobular carcinoma in situ (LCIS) prior to that screen.

We did not use the ‘family membership’ field in the Gail model source code designed for generating scores for groups of women (which would combine risk information from identified family members in the study group) as this information was unavailable in our data. Most race/ethnicity categories within the Gail model did not map to the ethnic profile of Australian women; as a best approximation, women who self-reported any Asian ethnicity were assigned to the Gail category ‘Asian-American’ (relabelled to ‘Asian’) and all other women to the category ‘White’ (labelled ‘Mixed’).

We generated Gail 5-year probability of breast cancer (‘scores’) for each woman and compared incident invasive breast cancer outcomes by quantile groups of risk (partitioned by group-level quintiles and/or deciles), for three age ranges (50–69/50–59/60–69 years). Hazard functions were censored to diagnosis (invasive or in situ), death, or 31 December 2016 (whichever occurred first). Quantile groups (i.e. quintiles and deciles) were generated for each age range analysed to reflect how the Gail model would assign women to risk groups if used on specific age groups. Receiver operating curves (ROC) were generated for outcomes against continuous Gail scores for women with a minimum follow-up period of 3 years. To compare observed and estimated diagnoses, we generated the Gail predicted probability of breast cancer for each woman for her observation period by linear interpolation between annual-year Gail estimates. Of note, the order of Gail scores does not change with the specified duration of future risk so that women would be ranked the same if we described 1-year, 5-year or 10-year risk. However, the expected number of cancers in this study are dependent on the follow-up time for each woman, so that women with the same rank of baseline risk but different observation periods (e.g. 3 years versus 6 years) would have a different probability of a cancer being observed during the follow-up period. We then summed these observation period-based probabilities for each Gail 5-year risk quantile group to generate the expected number of cancers within that group, and compared this with the observed number of cancers using chi-squared tests and ratios of expected to observed cancers (confidence intervals (CIs) calculated as for Constantino et al. [29]). Statistical tests used Stata 15 software (StataCorp, College Stations, TX, USA).

#### Reduced variable Gail models

**Fig. 1:**
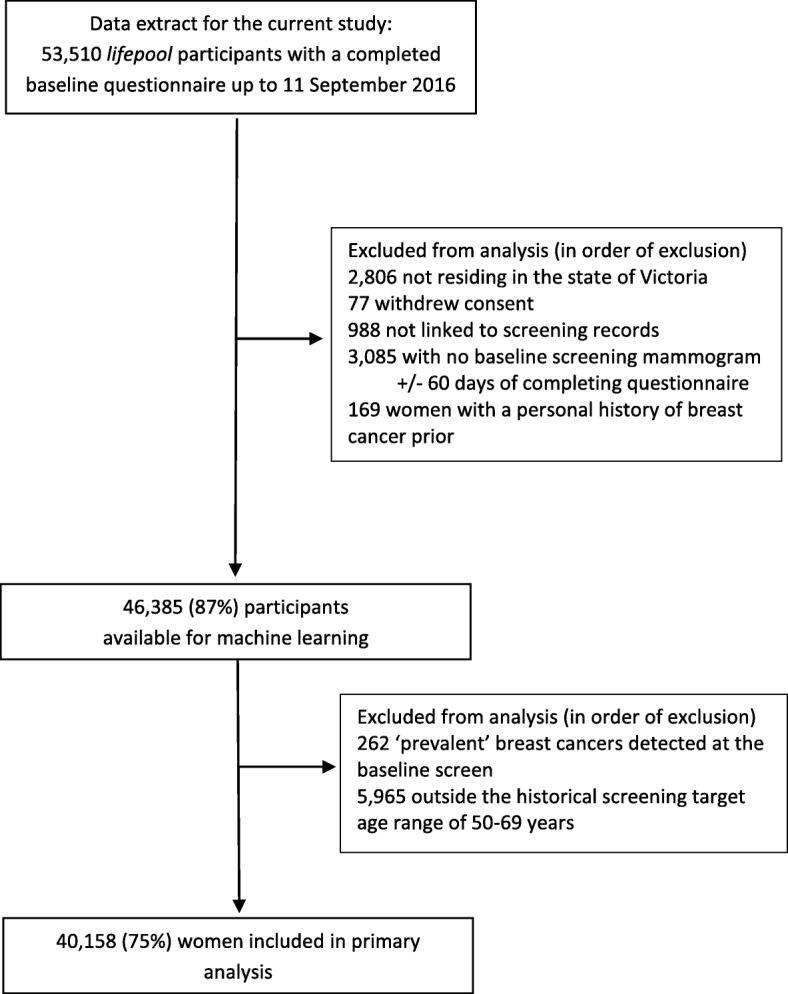
Flow chart demonstrating how the cohort used in analyses was derived from the original *lifepool* cohort

We evaluated Gail models using a reduced number of input variables, starting with the most important predictor of cancer risk in this cohort as identified using a machine learning approach. To maximise information to train and validate machine learning, we extended the dataset to all ages and women with invasive cancer diagnosed at the baseline mammogram (Fig. 1). The eight Gail variables (‘features’) were ranked using the feature importance function in XGBoost (version 0.72) implemented in Python (version 3.4). We conducted 100 extractions of training and test datasets. For each extraction, we randomly selected a test set (*N* = 6131) comprising a representative balance of cases (women who developed breast cancer) and controls (women who did not develop breast cancer) and a corresponding training set (*N* = 16,269) weighted to have a ratio of 1:9 cases to controls. The model was trained on each training dataset and validated on the corresponding test dataset, generating 100 ranks of variable importance which were then combined in a single ranking of variables according to the number of times each variable appeared in that ranking. Gail scores were calculated for each model by step-wise addition of variables according to that ranking (Models 1–8), with these scores then categorised into quantile groups and then evaluated under a hazards framework as for the whole model.

## Results

### Cohort characteristics

A total of 40,158 women (75% of the cohort) were included in our analyses. Major exclusions were: 2806 women who resided outside the state of Victoria at the time of completing their questionnaire because their subsequent diagnoses were unlikely to appear on Victorian screening and cancer registry records; 988 women who were not linked to screening records; 3085 women who did not have a baseline screening mammogram within 60 days of completing their questionnaire; and 169 women with a personal history of breast cancer prior to their reference screen. We excluded a further 262 women who had had a breast cancer diagnosis (205 invasive and 57 DCIS) at their reference screen, and 5965 women outside the historical BreastScreen target age range of 50–69 years at their reference screen for logistic regression analyses (however, these women were included in the machine-learning sample). No women remaining in the sample had a LCIS diagnosis at or prior to their reference screen. Additional exclusions are presented in Fig. 1.

During a median follow-up of 4.3 years, 564 women (1.4%) were diagnosed with invasive breast cancer (Table 1). The median time from the reference screen to diagnosis was 813 days (2.2 years), with a maximum of 5.3 years. Three women were diagnosed with incident LCIS (one with subsequent invasive breast cancer within the follow-up period), and 243 deaths from all causes were reported of which eight were due to breast cancer. Gail model variables for this group are described in Table 2. Women who developed invasive breast cancer were older at enrolment, more likely to have first-degree female relatives with breast cancer, and were more likely to have had a breast biopsy. Approximately 3% of all participants were of Asian ethnicity; however, it should be noted that women in the ‘mixed’ group were ethnically heterogeneous. Nearly all women (95%) attended screening during the follow-up period (Table 1).

**Table 1:** Summary characteristics of the *lifepool* participants by age groups

Characteristic	Age at reference screen (years)		
	50–69	50–59	60–69
Number of subjects (*N*)	40,158	20,216	19,942
Dates			
Reference screen (range)	1 Jul 2010 to 6 Oct 2014	1 Jul 2010 to 6 Oct 2014	1 Jul 2010 to 6 Oct 2014
Questionnaire completion (range)	1 Jul 2010 to 1 Oct 2014	1 Jul 2010 to 23 Sep 2014	31 Jul 2010 to 1 Oct 2014
Observation time, years (median, range)	4.3 (0.3–6.5)	4.3 (0.3–6.5)	4.3 (0.3–6.5)
Age			
Reference screen, years (median, range)	59 (50–69)	55 (50–59)	64 (60–69)
Diagnosis, years (median, range)	63 (51–73)	58 (51–64)	67 (61–73)
Reference screening round (median, range)	5 (1–18)	4 (1–18)	7 (1–18)
Diagnoses (invasive breast cancer)			
Number (%)	564 (1.4%)	244 (1.2%)	320 (1.6%)
Follow-up period			
Time from reference screen to diagnosis, days (median, range)	813 (28–1938)	818 (28–1714)	807 (73–1938)
Women screened during the follow-up period (*n*, %)	38,060 (95%)	19,141 (95%)	18,919 (95%)
Of women screened during the follow-up period, number of screens per 2 years of follow-up (median, range)	0.8 (0.3–2.0)	0.8 (0.3–2.0)	0.8 (0.3–1.9)

**Table 2:** Risk factors used to generate the Gail model scores among cases of women with invasive breast cancer and non-cases (i.e. women without invasive breast cancer), aged 50 to 69 years within the *lifepool* cohort^a^

Gail model variable	Group	Non-cases *n* = 39,594 (%)	Cases *n* = 564 (%)	*p* value^b^
Age at questionnaire (years)	50–54	8820 (22)	91 (16)	< 0.001
	55–59	11,152 (28)	153 (27)	
	60–64	10,968 (28)	158 (28)	
	65–69	8654 (22)	162 (29)	
Age at menarche (years)	≤ 11	6709 (17)	105 (19)	0.038
	12–13	19,995 (53)	300 (55)	
	≥ 14	11,224 (30)	143 (26)	
	Missing	1666	16	
Age at first live birth^c^ (years)	< 20	3468 (9)	57 (10)	0.22
	20–24	12,011 (30)	156 (28)	
	25–29	12,090 (30)	175 (31)	
	≥ 30	6413 (16)	111 (20)	
	Missing	2330	11	
	Nulliparous	3282 (8)	54 (10)	
Number of first-degree relatives (mother, sisters, daughters) who have had breast cancer	0 (or not reported)	30,531 (77)	390 (69)	< 0.001
	1	8067 (20)	158 (28)	
	2	933 (2)	16 (3)	
	3	61 (0)	0 (0)	
	4	2 (0)	0 (0)	
Breast biopsy	No	33,048 (86)	446 (82)	0.001
	Yes	5158 (14)	100 (18)	
	Missing	1388	18	
Number of breast biopsies	0	32,824 (87)	444 (82)	0.001
	1	3949 (10)	77 (14)	
	2	1040 (3)	21 (4)	
	Missing	1781	22	
Biopsy with atypical hyperplasia	No	1490 (88)	23 (92)	0.76
	Yes	209 (12)	2 (8)	
	Missing^d^	3459	75	
Race/ethnicity	Mixed^e^	38,428 (97)	555 (98)	0.059
	Asian	1166 (3)	9 (2)	

^a^The distribution of values for each variable is presented without inclusion of missing values

^b^Excluding missing. *P*-values for binary categories are from chi-square tests or Fisher’s exact test as appropriate; variables with three or more categories were assessed using a non-parametric test for trend (Stata ‘nptrend’)

^c^Data for assessment of this variable were not directly available; we used age at first full-term pregnancy for all women who had at least one live birth

^d^Missing shown only for women who responded ‘Yes’ to breast biopsy

^e^Other: women of non-Asian ethnicity

### Cancer incidence

**Fig. 2:**
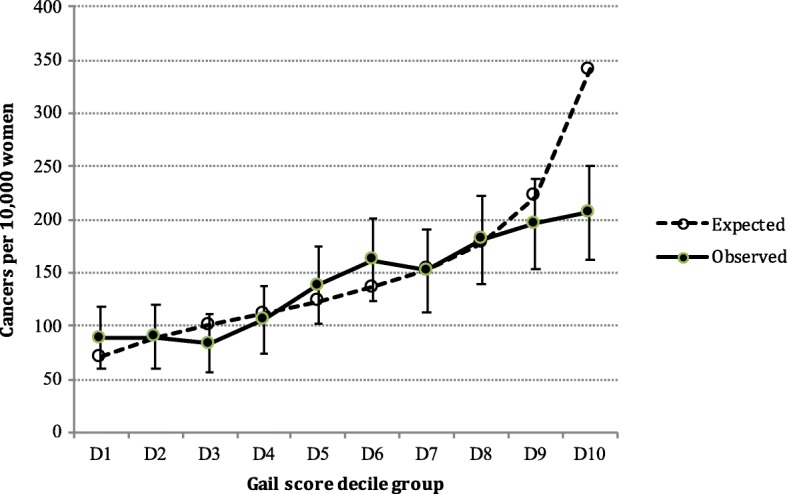
Expected and observed outcomes according to Gail scores generated by baseline questionnaires. Overall chi-squared test, *p* < 0.0001 (D1–D9 categories only; *p* = 0.57). D decile

Observed and expected diagnoses are shown as rates according to decile groups of Gail model-predicted 5-year risk in Fig. 2, with ratios of expected to observed invasive cancers (E/O) according to quantile groups of predicted 5-year risk shown in Table 3. Overall, the model was generally well calibrated with some evidence of over-prediction in women at the highest level of risk; 612 cases were predicted compared with 564 cases observed, corresponding to an expected-to-observed ratio of 1.09 (95% CI 1.00–1.18). Expected and observed outcomes by quintile groups differed significantly overall (χ^2^ = 23.0, *p* < 0.0001). E/O did not differ significantly for quantile groups Q1–Q4 and D9; however, the Gail model overestimated risk for women in decile group D10 (E/O 1.65, 95% CI 1.33–2.07), leading to a net overestimation in group Q5 (E/O 1.40, 95% CI 1.20–1.64). Similar patterns persisted within age groups 50–59 and 60–69 years (E/O 1.08, 95% CI 0.96–1.23, and 1.09, 95% CI 0.97–1.22, respectively).

**Table 3:** Comparison of expected and observed cases of invasive breast cancer, and hazard ratios for observed cases, according to Gail model predicted 5-year risk for all women by age group, and for group level risk quintiles (Q1 to Q5) and, within Q5, the upper two deciles of risk (D9 and D10)

Age (years)	Quantile group	Predicted 5-year risk (range)	No. of women	Observed (O) breast cancers	Expected (E) breast cancers	Person-years (PY)	O per 10,000 PY	E/0 (95% CI)^a^	HR (95% CI), p value (Q3 referent)	HR (95% CI), p value (Q1 referent)^b^
50–69	Q1	0.6–1.1%	8041	72	65	34,078	21	0.90 (0.71–1.15)	0.59 (0.44–0.79), *p* < 0.0001	Referent
	Q2	1.1–1.4%	8096	77	86	34,160	23	1.12 (0.89–1.42)	0.63 (0.47–0.84), *p* = 0.001	1.07 (0.78–1.48), *p* = 0.679
	Q3	1.4–1.7%	8124	122	105	34,264	36	0.86 (0.72–1.04)	Referent	1.70 (1.27–2.27), *p* < 0.0001
	Q4	1.7–2.3%	7902	132	131	33,254	40	0.99 (0.84–1.19)	1.11 (0.87–1.43), *p* = 0.388	1.89 (1.42–2.52), *p* < 0.0001
	Q5	2.3–22.0%	7995	161	225	33,690	48	1.40 (1.20–1.64)	1.34 (1.06–1.70), *p* = 0.014	2.28 (1.73–3.02), *p* < 0.0001
	D9	2.3–3.0%	3980	78	88	16,790	46	1.13 (0.91–1.43)	1.30 (0.98–1.73), *p* = 0.068	2.21 (1.61–3.05), *p* < 0.0001
	D10	3.0–22.0%	4015	83	137	16,900	49	1.65 (1.33–2.07)	1.39 (1.05–1.83), *p* = 0.022	2.35 (1.72–3.23), *p* < 0.0001
	Total	0.2–21.7%	40,158	564	612	169,445	33	1.09 (1.00–1.18)		
50–59	Q1	0.6–1.1%	4046	35	29	17,131	20	0.83 (0.60–1.19)	0.83 (0.53–1.30), *p* = 0.413	Referent
	Q2	1.1–1.4%	4054	33	38	17,167	19	1.14 (0.81–1.66)	0.78 (0.49–1.23), *p* = 0.283	0.94 (0.58–1.51), *p* = 0.797
	Q3	1.4–1.7%	4062	42	46	17,117	25	1.10 (0.81–1.52)	Referent	1.21 (0.77–1.89), *p* = 0.413
	Q4	1.7–2.3%	4013	67	58	16,917	40	0.87 (0.68–1.12)	1.61 (1.10–2.37), *p* = 0.015	1.95 (1.29–2.93), *p* = 0.001
	Q5	2.3–13.9%	4041	67	94	17,057	39	1.40 (1.10–1.80)	1.60 (1.09–2.35), *p* = 0.017	1.93 (1.28–2.90), *p* = 0.002
	D9	2.1–2.5%	2028	30	38	8567	35	1.28 (0.89–1.89)	1.43 (0.89–2.28), *p* = 0.138	1.72 (1.06–2.80), *p* = 0.029
	D10	2.5–13.9%	2013	37	55	8490	44	1.49 (1.08–2.12)	1.77 (1.14–2.76), *p* = 0.011	2.14 (1.35–3.40), *p* = 0.001
	Total	0.2–21.7%	20,216	244	264	85,388	29	1.08 (0.96–1.23)		
60–69	Q1	0.9–1.1%	4026	41	39	17,022	24	0.96 (0.71–1.34)	0.64 (0.43–0.95), *p* = 0.026	Referent
	Q2	1.1–1.4%	3992	55	48	16,833	33	0.88 (0.68–1.17)	0.87 (0.61–1.25), *p* = 0.458	1.36 (0.91–2.04), *p* = 0.134
	Q3	1.4–1.7%	4041	64	59	17,069	37	0.92 (0.72–1.19)	Referent	1.56 (1.06–2.31), *p* = 0.026
	Q4	1.7–2.3%	3946	74	75	16,573	45	1.02 (0.81–1.30)	1.19 (0.85–1.67), *p* = 0.302	1.86 (1.27–2.73), *p* = 0.0001
	Q5	2.3–22.0%	3937	86	126	16,560	52	1.47 (1.19–1.84)	1.40 (1.01–1.93), *p* = 0.044	2.18 (1.50–3.16), *p* < 0.0001
	D9	2.8–3.3%	1953	40	50	8192	49	1.24 (0.91–1.74)	1.31 (0.88–1.94), *p* = 0.182	2.04 (1.32–3.16), *p* = 0.001
	D10	3.3–22.0%	1984	46	77	8368	55	1.66 (1.25–2.27)	1.48 (1.01–2.16), *p* = 0.042	2.31 (1.52–3.53), *p* < 0.0001
	Total	0.2–21.7%	19,942	320	348	84,057	38	1.09 (0.97–1.22)		

CI confidence interval, D, decile, HR hazard ratio, Q quintile

^a^ Chi-squared test across Q1–Q5 (O vs E) were: χ^2^= 23.0, *p* = 0.0001 for women 50–69 years old; χ^2^ = 11.0, *p* = 0.0262 for women 50–59 years old; and χ^2^ = 14.4, *p* = 0.0063 for women 60–69 years old

^b^ Log rank tests for trend across hazard functions Q1–Q5 were χ^2^ = 52, *p* < 0.0001 for women 50–69 years old; χ^2^ = 20, *p* < 0.0001 for women 50–59 years old; and χ^2^ = 21, *p* < 0.0001 for women 60–69 years old

Hazards ratios for invasive cancer incidence by Gail model 5-year risk quantile groups are shown in Table 3. Compared with women with a median-level risk (Q3), women in the lowest two quintile groups had a 37–41% decreased risk of invasive cancer (Q1 vs Q3: 0.59, 95% CI 0.44–0.79, *p* < 0.001; Q2 vs Q3: 0.63, 95% CI 0.47–0.84, *p* = 0.001) and those above the highest quintile had a 34% increased risk (Q5 vs Q3: 1.34, 95% CI 1.06–1.70, *p* = 0.014).

When compared with women with the lowest scores (Q1), the risk of invasive cancer increased by quintile group, being statistically significant for group Q3 and above. Group Q5 had a 2.28-fold increased risk compared to Q1 (hazard ratio (HR) 2.28, 95% CI 1.73–3.02, *p* < 0.0001). Hazard functions followed a significant trend across risk groups (χ^2^ = 52, *p* < 0.0001). The ROC area under the curve (AUC) using continuous Gail scores was 0.59 (95% CI 0.56–0.61) for women aged 50–69, 0.59 (95% CI 0.55–0.62) for women aged 50–59, and 0.57 (95% CI 0.54–0.60) for women aged 60–69 years.

### Reduced Gail model

**Fig. 3:**
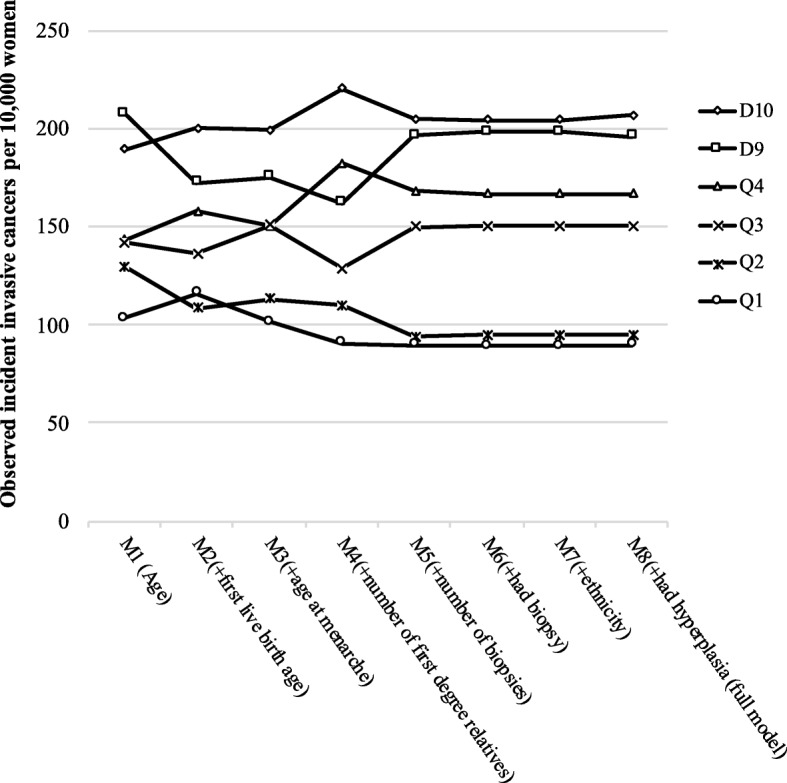
Observed incident cancers per 10,000 women according to quantile groups for the reduced BCRAT models (M1–8) assessed. M model, Q quintile

Machine learning models ranked the importance of Gail model variables as ordered in Table 4 (age being the most important). Most variables were consistently ranked for the 100 runs, except for ‘first live birth age’ and ‘age at menarche’ which exchanged places having a 62% frequency of ranking in second and third positions, respectively. Hazard ratios for each quintile group were found to vary as the first four variables were progressively added (Models 1–5) but changed little with the addition of further variables (Models 6–8); Model 5 (incorporating number of biopsies) led to a more accurate ranking of observed outcomes than Models 1–4 (Fig. 3). For Model 5, women in group Q5 had a 2.28-fold higher risk of developing invasive breast cancer compared with women in Q1 (95% CI 1.73–3.01) (Table 4). Of note, when the number of first-degree relatives was added (Model 4), the expected values increased greatly in the upper decile but the observed values did not rise to match (E/O for D10 was 0.99–1.03 for Models 1–3, then 1.51–1.66 for Models 4–8). Therefore, Model 4 appears comparable to the full Gail model in terms of stratifying women into risk groups.

**Table 4:** Hazard ratios for incident invasive breast cancer in women 50–69 years old, according to reduced Gail model (5-year risk) quintiles and area under the curve (AUC) for each model using continuous Gail scores

Gail Subset (model)	Frequency in rank (%)	Risk score quintile group (5-year risk)							Test for trend (Q1 to Q5)^a^	AUC (95% CI)
		Q1	Q2	Q3	Q4	Q5	D9	D10		
Age (M1)	100	Referent	1.25 (0.95–1.64), *p* = 0.112	1.37 (1.03–1.83), *p* = 0.029	1.38 (1.06–1.81), *p* = 0.018	1.96 (1.51–2.56), *p* < 0.001	2.06 (1.52–2.79), *p* < 0.001	1.85 (1.33–2.58), *p* < 0.001	χ^2^ = 23, *p* < 0.0001	0.56 (0.53–0.58)
+ first live birth age (M2)	62	Referent	0.94 (0.70–1.26), *p* = 0.686	1.18 (0.90–1.54), *p* = 0.229	1.38 (1.05–1.80), *p* = 0.02	1.64 (1.27–2.12), *p* < 0.001	1.50 (1.09–2.06), *p* = 0.012	1.77 (1.32–2.38), *p* < 0.001	χ^2^ = 21, *p* < 0.0001	0.56 (0.54–0.58)
+ age at menarche (M3)	62	Referent	1.12 (0.83–1.51), *p* = 0.458	1.50 (1.13–1.98), *p* = 0.004	1.49 (1.13–1.97), *p* = 0.005	1.89 (1.44–2.47), *p* < 0.001	1.76 (1.28–2.43), *p* = 0.001	2.01 (1.48–2.73), *p* < 0.001	χ^2^ = 26, *p* < 0.0001	0.56 (0.54–0.59)
+ number of first-degree relatives (M4)	84	Referent	1.22 (0.90–1.67), *p* = 0.200	1.43 (1.06–1.93), *p* = 0.019	2.05 (1.55–2.71), *p* < 0.001	2.15 (1.63–2.84), *p* < 0.001	1.82 (1.30–2.54), *p* < 0.001	2.49 (1.82–3.39), *p* < 0.001	χ^2^ = 45, *p* < 0.0001	0.58 (0.56–0.60)
+ number of biopsies (M5)	79	Referent	1.06 (0.77–1.46), *p* = 0.730	1.69 (1.27–2.27), *p* < 0.001	1.91 (1.43–2.54), *p* < 0.001	2.28 (1.73–3.01), *p* < 0.001	2.22 (1.61–3.06), *p* < 0.001	2.34 (1.71–3.21), *p* < 0.001	χ^2^ = 52, *p* < 0.0001	0.59 (0.56–0.61)
+ had biopsy (M6)	91	Referent	1.07 (0.78–1.48), *p* = 0.672	1.70 (1.27–2.27), *p* < 0.001	1.90 (1.43–2.52), *p* < 0.001	2.29 (1.74–3.02), *p* < 0.001	2.24 (1.63–3.08), *p* < 0.001	2.33 (1.70–3.20), *p* < 0.001	χ^2^ = 52, *p* < 0.0001	0.59 (0.56–0.61)
+ ethnicity (M7)	98	Referent	1.07 (0.78–1.48), *p* = 0.672	1.70 (1.27–2.27), *p* < 0.001	1.90 (1.43–2.52), *p* < 0.001	2.29 (1.74–3.02), *p* < 0.001	2.24 (1.63–3.08), *p* < 0.001	2.33 (1.70–3.20), *p* < 0.001	χ^2^ = 52, *p* < 0.0001	0.59 (0.56–0.61)
+ had hyperplasia (full model) (M8)	100	Referent	1.07 (0.78–1.48), *p* = 0.679	1.70 (1.27–2.27), *p* < 0.001	1.89 (1.42–2.52), *p* < 0.001	2.28 (1.73–3.02), *p* < 0.001	2.21 (1.61–3.05), *p* < 0.001	2.35 (1.72–3.23), *p* < 0.001	χ^2^ = 52, *p* < 0.0001	0.59 (0.56–0.61)

Values are shown as hazard ratio (95% CI), *p* value

CI confidence interval, D decile, Q quintile

^a^ Tests for trend for each model across hazard functions for risk score groups Q1 to Q5

## Discussion

Comparing outcomes arising within a maximum of 6.5 years follow-up, we found that women aged 50–69 years within the highest quintile of Gail risk scores (Q5) had more than double the risk of invasive breast cancer compared with women in the lowest quintile (Q1). Compared with women in the median-risk group (Q3), Q1 had a 40% reduced risk and Q5 a 34% increased risk of incident invasive breast cancer. This suggests that the existing Gail model is suitable for assigning women into groups at significantly different risk of invasive breast cancer in the 5 years following a negative screen.

We found good overall agreement between expected and observed cases of invasive breast cancer, confirming absolute risk estimates over an average of 4.3 years of follow-up except for women in the upper decile of Gail scores; while these women were appropriately classified as the highest-risk group, their absolute Gail risk scores overestimated the observed outcomes (Fig. 2 and Table 3). This may be due to the exclusion of higher-risk women such as women with cancer diagnosed at the first-round or other prior screening episodes and/or women who attend high-risk services rather than BreastScreen due to a family history or identified increased genetic risk of breast cancer. This latter theory is supported by the increase in expected cancers in group D10 with the addition of family history to the reduced Model 5, without a concomitant increase in the observed number of cancers in that group. Therefore, using the Gail model in this population is expected to rank women well into the quantile groups examined; however, for women assigned to the highest decile of risk (> 3% estimated 5-year risk) a more detailed risk assessment or alternative models incorporating additional family history information might be considered, such as that proposed by Pfeiffer et al. [30]. The current Gail model does not incorporate high-risk gene mutations such as BRCA1/2; in Australia, such women are referred to more intensive surveillance outside the BreastScreen programme.

Of note, the ethnicity variable was ranked with low importance in our machine learning models, reflecting poor correspondence between Australian ethnicity groups and the Gail ‘race’ variable values. A modified ethnicity variable suited to the local population may improve risk classification, as breast cancer risk does differ by country of birth in the Australian population (for example, age-standardised rates of 71 (95% CI 67–76) per 100,000 women born in north-east Asia compared with 120 (107–133) per 100,000 women born in the USA or Canada) [31].

Using machine learning, a reduced model resulted in hazard ratios comparable to the full Gail model, suggesting that a simplified model (e.g. limited to age, first live birth age, age at menarche, number of first-degree female relatives with breast cancer, and possibly history of biopsy) could be equally effective in this population while saving significant effort and resources. Unsurprisingly, the stepwise addition of the variables ‘had biopsy’ made little difference since the number of biopsies was already included. The ethnicity variable would hold more value if the Gail model was modified to suit Australian ethnicity categories.

The modest discriminatory accuracy of the Gail model (AUC = 0.59) is consistent with a recent meta-analysis of European validation studies (pooled AUC =0.58) [32], confirming that risk information should be conveyed clearly and carefully to ensure that it is understood to apply to group-level rather than individual-level risk. However, group-level estimates such a 5-year risk of less than 1% for women in the lowest quintile versus more than 3% for women in the upper decile (Table 3) are meaningful for group-level health advice and interventions, such as the potential value of more personalised screening protocols targeted to specific risk groups.

This study has various strengths. Analyses are based on data from a large prospective cohort of actively screened participants, with questionnaires completed during 2010–2014 and outcomes recorded up to end 2016, and therefore results are highly relevant to contemporary screening populations and programmes. Cancer outcomes were identified through direct linkage with cancer registrations, and screening histories by direct linkage with the screening programme. We accounted for censoring by using hazards models, and we report outcomes for groups based on quintile and decile values to demonstrate potential applications for this tool not only to identify women at very high risk of breast cancer but also to identify women at medium and reduced risk of breast cancer.

Our study has several limitations. Firstly, we did not have records of cancers diagnosed outside the state of Victoria, although these are likely to be few. Secondly, we did not have complete death records. Based on Australian deaths data [33] (average death rates for 2010–2012 by 5-year age group applied to observed person-years to the end of 2016), the expected number of all-cause deaths in this cohort is approximately 724 (versus 243 recorded deaths). Our ‘expected’ cancers will therefore be slightly overestimated due to overestimated exposure time to risk of breast cancer for women without a cancer registered in Victoria. This may help explain why the expected number of cancers exceeded the observed number. However, because other-cause death is unlikely to be strongly associated with the Gail model within the age group examined, confounding would be minimal. Another limitation relates to the generalisation to the whole screened population; our sample is drawn from BreastScreen participants who consented to participate in the *lifepool* cohort and these women may be more willing and/or able than other BreastScreen participants to provide the information required for the Gail model.

This study contributes to the international body of evidence on the validity of the Gail model as well as providing information on the model’s applicability in a population breast screening setting. Although several validation studies of Gail model predictions on prospective cohorts have been conducted [32], limited validation studies have been performed on women attending routine breast cancer screening [14, 34–38]. This is the first validation study applied to a population of breast cancer screening participants in Australia.

As appropriate for validating a predictive tool, our analysis excluded from our study group women with a breast cancer diagnosis at or prior to their ‘baseline’ *lifepool* recruitment screen; it is possible that the observed rates of cancer would be slightly different if the risk tool was applied to all women at first-round screening, or if the risk tool was applied to the general population (e.g. through general practice). Since its inception, the Gail model has been modified to account for the variation in breast cancer risk observed in various populations [23–25]. Risk predication can be improved by combining the Gail model with mammographic density [21, 34] and genetic factors [27, 38]. Future work by our group will extend the use of machine learning methods to generate breast cancer risk prediction models based on *lifepool* cohort data, optimally combining clinical, genetic, mammographic density, and behavioural risk factors. We will also report outcomes for younger and older women, by mode of detection (screen, interval or other), and incidence of DCIS as the *lifepool* cohort matures.

## Conclusions

The findings from this study indicate that the Gail model, or a simplified version of this model, is an effective tool for stratifying active breast cancer screening participants aged 50–69 years to groups according to risk of invasive breast cancer diagnosed up to 5 years following risk assessment.

## Additional file

Additional file 1: **Table S1.** Details of data provided by *lifepool* for this study. (DOC 33 kb)

## Abbreviations

AUC: Area under the curve
CI: Confidence interval
D: Decile
DCIS: Ductal carcinoma in situ
LCIS: Lobular carcinoma in situ
M: Model
Q: Quintile
