# Supplementary material for: Prospective validation of the NCI Breast Cancer Risk Assessment Tool (Gail Model) on 40,000 Australian women
Source: Breast Cancer Res. 2018 Dec 20;20:155. doi: 10.1186/s13058-018-1084-x (PMC6302513; doi:10.1186/s13058-018-1084-x)
Supplement: Supplementary file 1 — Table S1. Details of data provided by lifepool for this study. (DOC 33 kb) [file 13058_2018_1084_MOESM1_ESM.doc]

| **Source** | **Data type** | **Inclusions** | **Date data sent to *lifepool*** |
| --- | --- | --- | --- |
| VCR | Cancer diagnoses | Data complete to 31 Dec 2016 with some diagnoses processed for 2017 | 27 March 2018 |
| VCR | Death notifications | For women with any cancer diagnosis, notifications of deaths in Victoria occurring up to 31 Dec 2016 and some deaths for 2017 (see above), and interstate deaths (considered complete to end of 2015) | 27 March 2018 |
| BSV | Screening episodes | Episodes occurring up to 27 June 2017 | 19 June 2017 |
| BSV | Screen detected cancers | Cancers detected up to 9 Nov 2016 | 19 June 2017 |
| BSV | Interval cancers* | Complete for screening episodes up to end 31 December 2013 (latest cancer diagnosis date 9 November 2016. | 19 June 2017 |

BSV: BreastScreen Victoria; VCR: Victorian Cancer Registry: VCR.

* Interval cancers: diagnosed within 27 months of a negative screen. We use this definition to help identify ‘baseline’ screen-detected cancers for exclusion.
